# A Central Slip Injury in a Professional Basketball Player

**DOI:** 10.7759/cureus.35197

**Published:** 2023-02-19

**Authors:** Eric Chun-Pu Chu, Andy Fu Chieh Lin, Sharon Mok, Shun Zhe Piong, Gabriel Ng

**Affiliations:** 1 Chiropractic Department, EC Healthcare, Hong Kong, HKG

**Keywords:** extensor carpi ulnaris subluxation, mallet fingers, boutonniere deformity, central slip injury, chiropractic

## Abstract

Hand injuries are extremely prevalent in sports such as ball sports. Delayed diagnosis or improperly managed injuries have the potential to prolong the athlete’s return to the competition and permanently damage their abilities. We report the case of a 35-year-old male professional basketball player who presented to a chiropractor with pain and swelling of the right fifth finger two days after a basketball game. The injury occurred when a player passed the basketball and the ball hit his fifth finger "head-on". Examination revealed enlargement of the middle joint of the right fifth finger and a boutonniere deformity. He was diagnosed with a central slip injury using magnetic resonance imaging (MRI). Since there are no standard treatment guidelines for central slip injuries, multi-model chiropractic therapy was applied to the injury and adjacent sites. The patient returned to the game by the end of the third week and fully recovered within six weeks. Chiropractors must understand how to best guide athletes’ clinical management of these injuries, given the inherent need for immediate and complete recovery.

## Introduction

Hand injuries are extremely prevalent in ball sports, and ligament and tendon injuries are among the leading causes of hand and finger deformities [1]. Common causes of central slip injury in athletes include direct blunt trauma and penetrating injury to the phalanges. The detachment and disruption of the extensor digitorum communis (EDC) tendon over the central slip can lead to a boutonniere deformity [2]. This deformity is characterized by the pathological flexion of the proximal interphalangeal (PIP) joint and hyperextension of the distal interphalangeal (DIP) joint [1]. The deformities and signs of a central slip injury can appear immediately after trauma.

However, owing to the lack of an open wound and the absence of radiological abnormalities, central slip injury is frequently misdiagnosed [2]. Chiropractors are healthcare professionals specializing in the treatment of musculoskeletal diseases. Although they rarely encounter patients with fractured bones or ruptured tendons [3], chiropractors who specialize in sports medicine might take care of sports injuries very often. This case report highlights the role of chiropractors in the diagnosis and treatment of central slip injuries. We addressed the challenges in the diagnosis and clinical management of central slip injuries in athletes.

## Case presentation

A 35-year-old male professional basketball player presented to a chiropractor with pain and swelling in the right fifth finger after a basketball game. He was right-hand dominant, and the pain was located on the top of the middle joint of the right fifth finger for two days. The injury occurred when a player passed the basketball and it hit his fifth finger "head-on". The patient was unable to straighten his fingers. The pain was rated 5/10 on a numeric pain scale, and ice and over-the-counter anti-inflammatory medications were administered to alleviate symptoms. He was a nonsmoker and social drinker and denied a family history of hand injuries or major pathologies.

**Figure 1:**
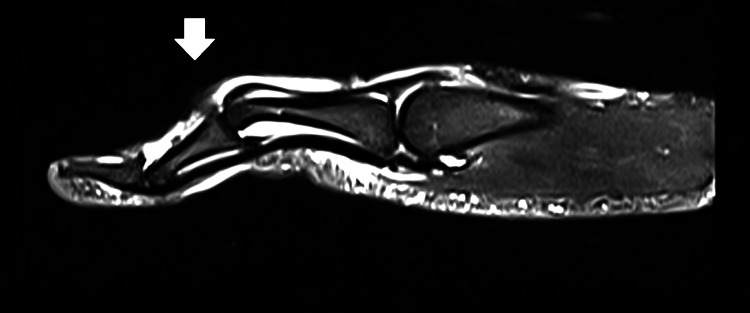
Magnetic resonance image of the fifth metacarpal joint Imaging revealed a tear of the central slip of the extensor tendon (arrow), along with a boutonniere deformity and volar subluxation of the middle phalanx at the proximal interphalangeal joint.

Examination revealed enlargement of the middle joint of the right fifth finger and a boutonniere deformity. The proximal interphalangeal (PIP) joint could not be straightened and the distal interphalangeal (DIP) joint could not be bent. The patient was unable to grasp and had a loss of motion while extending the PIP joint and hyperextension of the DIP joint. Palpation revealed hypertonicity of the right wrist extensors and right elbow flexors. The motor strength of the right wrist extension was rated 4 out of 5 because of pain, and all sensory and reflexes of the upper extremity were unremarkable. Orthopedic examinations revealed positive modified Elson test and Boyes test results for central slip injury. According to the patient history and orthopedic findings, the chiropractor’s initial differential diagnosis included central slip injury, phalanx fractures, rheumatoid arthritis, finger arthritis, and tendinopathy. Magnetic resonance imaging (MRI) revealed a tear in the central slip of the extensor tendon (arrow), along with a boutonniere deformity and volar subluxation of the middle phalanx at the proximal interphalangeal joint (Figure 1). Based on the patient’s history, physical examination, and radiological results, he was confirmed to have a central slip injury.

**Figure 2:**
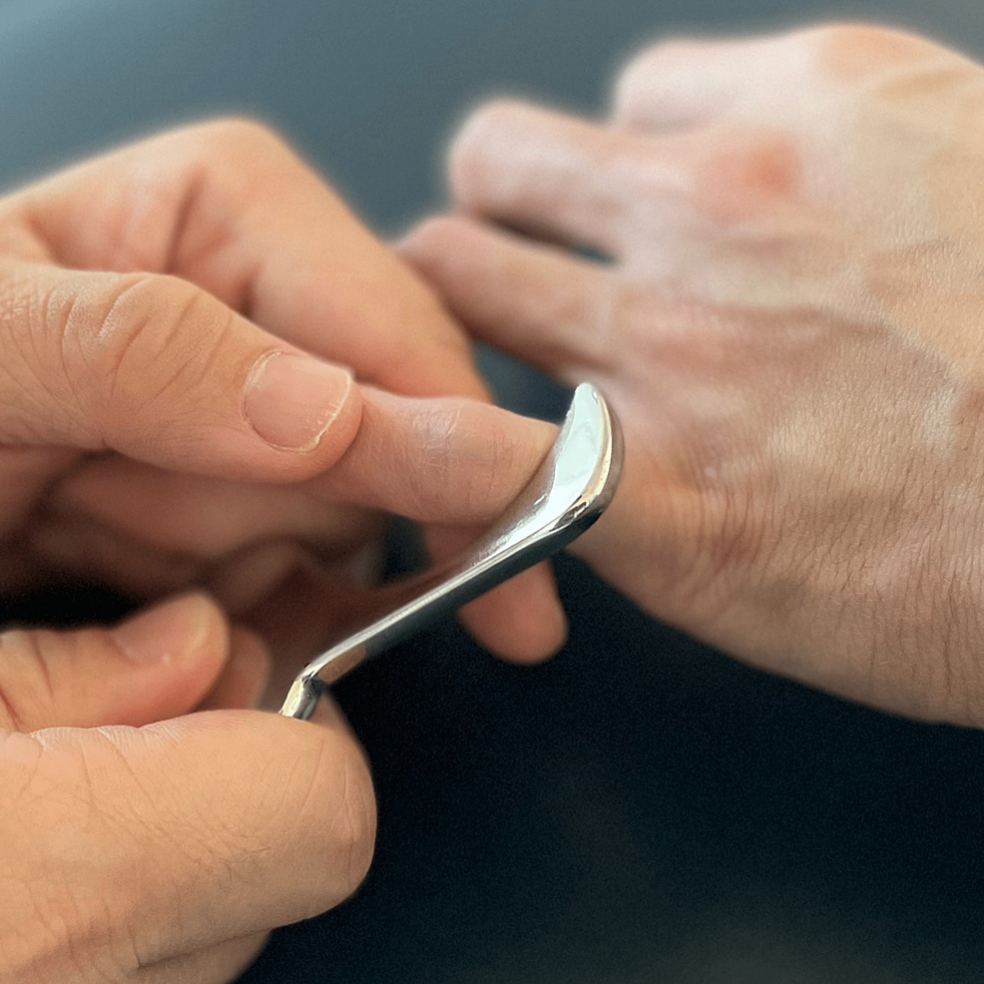
Instrument-assisted soft tissue mobilization Instrument-assisted soft tissue mobilization employs shear force to pull the layers of fascia in the fingers. The shear force breaks up the scar tissue between the layers and stimulates healing. As the instrument glides across the fibrotic areas of the soft tissue, the injury may feel rough or sandpaper-like, accompanied by redness and mild swelling. Treatment typically lasts five minutes. The patient usually reports an immediate difference after treatment.

**Figure 3:**
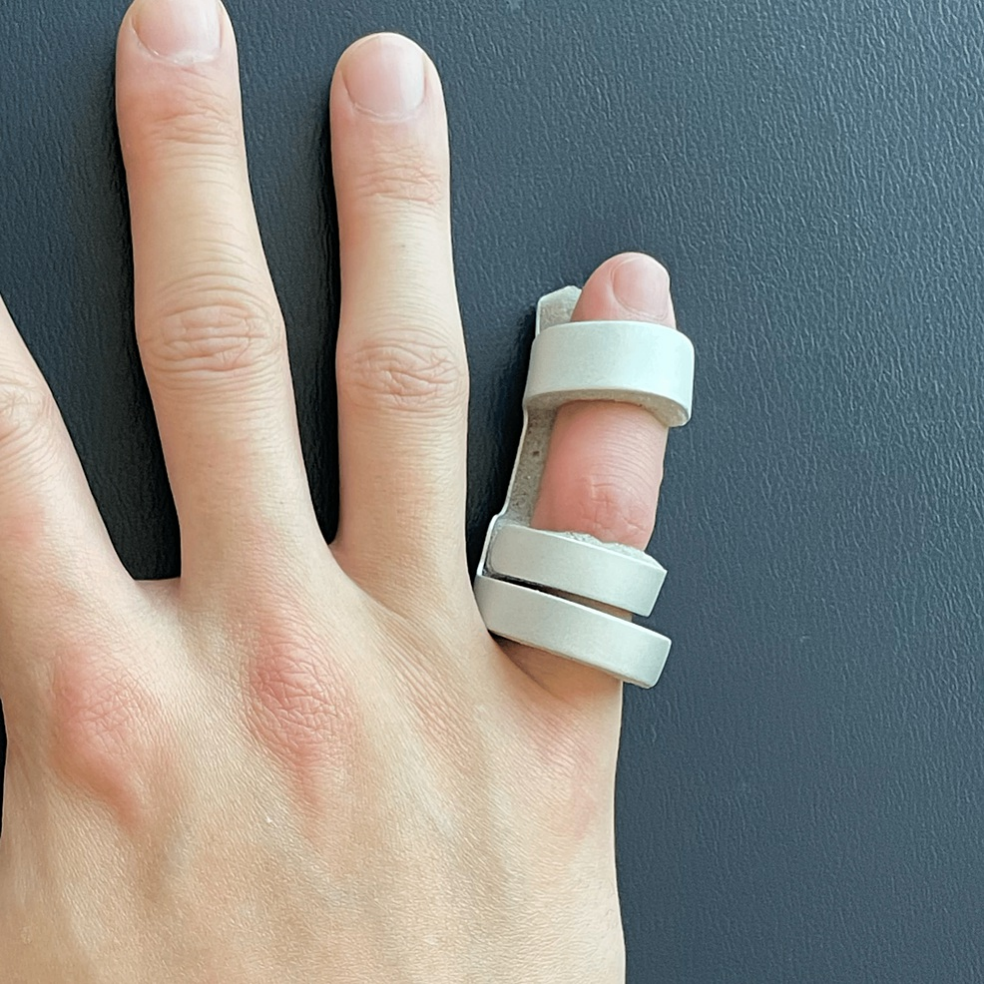
Finger splint For six weeks, the patient wore a splint on the fifth phalange to keep the PIP joint straight. This enables the tendon to repair in an optimal position. PIP: proximal interphalangeal

**Figure 4:**
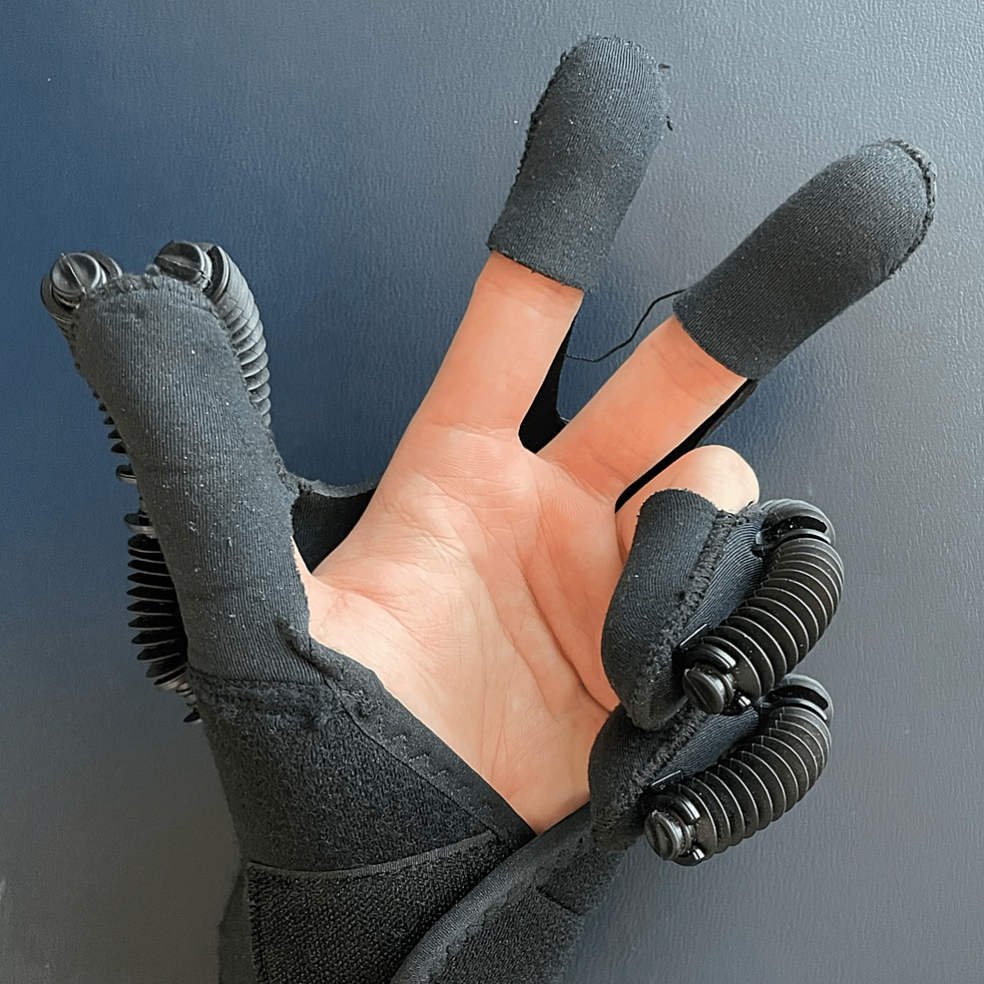
Robotic glove A robotic glove was provided to aid in home-based rehabilitation by facilitating repetitive stretching exercises to improve the finger’s rigidity and restore motion. The glove has inflatable chambers that flex and extend the phalanges gently, providing the necessary stretching and repetitive exercise to restore lost hand function.

The objective of chiropractic treatment is to heal the central slip and correct the finger deformity. Despite needing to return to the game in three weeks, the patient chose not to take medication due to their compliance with the sport's regulations. Thermal ultrasound therapy was applied to the PIP joint; joint manipulative therapy was administered to the radiocarpal joint, metacarpophalangeal joint, and DIP of the right hand; and instrument-assisted soft tissue mobilization was applied on the lateral sides of the PIP joint (Figure 2), extensor of the right wrist, and flexors of the right elbow. The treatment schedule was set to twice a week. A finger extension splint was prescribed for home use to straighten the finger and allow the tendon to heal back down to the bone for six weeks (Figure 3). Daily DIP joint flexion exercises with the PIP joint in extension were instructed to stimulate retraction of the posterior tendons and diminish the boutonniere deformity. By the end of the third week, despite the finger's typical rigidity, the swelling had disappeared, and the joint stiffness was minimal. After removing the splint, the joint frequently required some time to become flexible again; however, the patient was able to compete with the same performance. Joint manipulation therapy was administered to the PIP joint once weekly to reduce stiffness. By the end of the sixth week, his pain had resolved and finger motion had fully recovered. In addition to the previous treatments, a robotic glove was provided for an additional two weeks to perform hand exercises (Ober, China) (Figure 4). The patient wore the glove for 30 min per day at home. By the eighth week, the patient reported a complete resolution of symptoms.

## Discussion

Athletes' hands can sustain a variety of acute and cumulative damages depending on their sport [4]. Two percent of all hand injuries requiring medical attention are central slip injuries to the extensor tendon [5]. As central slip injuries frequently occur with concomitant hand injuries, they frequently exacerbate tendon injuries in the neighboring fingers, a phenomenon known as the quadriga phenomenon [6]. Because of the complexity of the extensor, an in-depth understanding of its anatomy can aid in both diagnosis and treatment [7]. Untreated or improperly managed injuries have the potential to result in persistent disability [7].

A clinical diagnosis of hand ligament injuries can be challenging. An analysis of patients with closed central slip injuries revealed that 60% had previously visited general practitioners or emergency rooms. However, 80% of patients had a delayed diagnosis of the injury [2]. Although orthopedic tests can assist clinicians in diagnosing central slip injuries [2], due to the absence of an open wound or radiographic abnormality, the injuries are often neglected or misdiagnosed [2]. Magnetic resonance imaging (MRI) is an accurate method for evaluating the pathophysiology and anatomy of the fingers and for diagnosing sports-related tendon and ligament injuries of the hand [1].

Chiropractors play an important role in treating sports injuries, as they often collaborate with other healthcare professionals to ensure that the athletes receive the best possible care, and they may also provide physical therapy if needed. Injuries to the extensor tendons of the hand in elite athletes can prolong their return to competition and have a permanent impact on their abilities [7]. This case illustrates the case of an elite athlete immediately diagnosed by a chiropractor using MRI. However, evidence addressing the treatment regimen for central slip injuries is inconsistent and unsupported by a systematic study comparing the outcomes of conservative and surgical care [8]. There are no standard treatment guidelines for central slip injury [5,9]. After searching the Index to Chiropractic Literature, Google Scholar, and PubMed, we were unable to identify any published cases of patients managed by a chiropractor with a diagnosis of a central slip injury as of February 8, 2023. Chiropractors need to understand how to best guide an athlete's clinical management given the inherent need for immediate and complete recovery. Treatment plans for hand injuries require individualized adaptation depending on the athlete's needs during the current competition, immediate clinical management of the injury, and future goals of the athlete [4]. Surgical treatment was considered only if the conservative treatment failed [5].

In contrast to other treatments mentioned in the literature, such as pain medication, splint and rest, and surgery [9], the treatment for central slip damage in this situation is a multimodal intervention at the injured and adjacent sites. Unlike traditional medical treatment utilizing medications and modalities such as electrical stimulation and ultrasound, an alternative conservative option was presented because the patient, a professional athlete, desired to refrain from taking medications. Given that the patient experienced pain alleviation soon after the therapy and adverse events related to chiropractic treatment are very rare [10], we hypothesized that the combination of adjacent joint manipulation and soft tissue mobilization contributed to his quick recovery. We believe that the relief achieved through joint manipulation was due to the restoration of normal joint movement in the periphery, which may have been impeded by injury or damage. Additionally, the mobilization of soft tissues with the aid of an instrument may reduce nociceptive signals and accelerate healing [11].

## Conclusions

Misdiagnosis of central slip injuries of the fingers in professional athletes can delay their return to competition, resulting in significant hand deformities. Due to the lack of evidence-based procedures, surgical treatment is not recognized as the gold standard. Chiropractors must understand how to best guide athletes’ clinical management of these injuries, given the inherent need for immediate and complete recovery.
